# Medicine Beyond Machines: Viewpoint on the Art of Thinking in the Age of AI

**DOI:** 10.2196/76669

**Published:** 2025-10-09

**Authors:** Isabella B B Ferreira, Rodrigo C Menezes, Luis Cláudio L Correia, Bruno B Andrade

**Affiliations:** 1 Departamento de Pós-Graduação, Escola Bahiana de Medicina e Saúde Pública, Salvador, Brazil; 2 Laboratório de Pesquisa Clínica e Translacional, Instituto Gonçalo Moniz, Fundação Oswaldo Cruz, Rua Waldemar Falcao, 121, Candeal, Salvador, 40296-710, Brazil, 55 71999721511

**Keywords:** artificial intelligence, AI, critical thinking, medicine, medical education

## Abstract

The widespread adoption of large language models is increasingly shaping clinical decision-making by altering how physicians engage with data and reasoning. While these tools enhance diagnostic capacity, streamline workflows, and support learning, their misuse may diminish critical, contextual, and humanized thinking, reducing physicians to passive validators of algorithmic outputs. This paper explores the evolution of medical cognition and proposes strategies for integrating artificial intelligence in ways that preserve cognitive autonomy, such as structuring information, reducing bias, and strengthening metacognition. We argue that artificial intelligence should serve as a “cognitive stethoscope,” a tool that refines reasoning without compromising its essence.

## Introduction

Before the advent of antibiotics, anesthetics, or hard technologies, medicine was built on the pillars of care and curiosity. Over time, this innate curiosity became intertwined with innovation, driving continuous improvement. Technology has since expanded human capabilities far beyond biological limits, reconfiguring the way in which we diagnose, treat, and even think.

Now, we stand at the threshold of another technological leap: the incorporation of artificial intelligence (AI) into clinical practice, particularly through large language models (LLMs) [1]. These tools go beyond providing information; they analyze patterns, process data, and suggest diagnoses and therapeutic approaches, fundamentally reshaping the relationship between physicians and technology. In this viewpoint, we argue that, unlike earlier innovations, which extended physicians’ senses without altering how they think, LLMs act directly on cognition itself. Used passively, they risk narrowing physicians’ judgment; used deliberately, they may serve as a “cognitive stethoscope,” refining clinical reasoning without supplanting it.

AI encompasses a broad field of systems that mimic human cognitive functions. Among its most influential branches is machine learning (ML), where algorithms learn from data to identify patterns and make predictions without explicit programming. A modern subset of ML, deep learning (DL), uses neural networks with multiple layers to detect complex, abstract representations, especially useful in tasks such as image or speech recognition. LLMs, in turn, are a specialized form of DL model trained on vast textual datasets, enabling applications such as clinical summarization, triage, and decision support [2].

The clinical potential of these tools is undeniable. However, their growing presence raises a fundamental question: to what extent does AI enhance medical cognition, and to what extent might it undermine it? Early studies suggest that, when integrated as a complement rather than a replacement, AI can improve diagnostic accuracy and efficiency without eroding independent reasoning [3]. Still, its impact depends less on raw capability than on how it is integrated into practice, an unsettled and increasingly urgent challenge.

However, medicine is more than a probabilistic exercise. Clinical decision-making is a complex, multidimensional art form that relies on judgment, inference, and human perception, aspects that algorithms have yet to replicate. In this paper, we explore the evolution of medical cognition and what distinguishes human thought from AI’s role in clinical practice, focusing on AI’s influence on diagnostic reasoning and therapeutic decision-making, while also considering its implications for education and workflow. Our goal is to orient physicians and medical students toward a deliberate, critical engagement with AI.

## Scientific Revolution and the Transformation of Medical Thinking

Before modern science, medicine was upheld by experimentation, beliefs, and individual observations. Diseases were attributed to miasmas or humoral imbalances, and treatments rested more on assumption than evidence [4]. Then, science began to unveil the invisible. In the 19th century, John Snow mapped London’s cholera outbreaks, tracing the source to a contaminated water pump. His work demonstrated that patterns hidden in chaos could guide medical reasoning, laying the foundation for modern epidemiology [5].

With the discovery of pathogenic microorganisms, medicine shifted from speculation to specificity [6]. Diseases such as tuberculosis were no longer seen as a “tragic fate” but as conditions caused by identifiable agents such as *Mycobacterium tuberculosis* [7]. This marked a new era in which tools such as the stethoscope, laboratory assays, and imaging technologies expanded clinical perception and enhanced our senses, allowing clinicians to hear internal sounds, decode biochemical markers, and visualize organs and tissues.

As medicine evolved, new technologies and interventions were evaluated through rigorous scientific methods. Intuition gave way to evidence, to randomized clinical trials and systematic reviews, transforming care from trial and error into a practice grounded in probabilities and measurable outcomes. This shift produced landmark successes; for example, antiretroviral therapy dramatically reduced mortality among patients with AIDS. Initially facing a 5-year mortality rate of 80% to 90%, individuals living with HIV now approach the life expectancy of the general population [8].

At the same time, medicine has become more complex. Where once it was clear when to administer insulin, drain an abscess, or amputate a gangrenous limb, today’s clinicians must navigate subtler trade-offs involving marginal benefits, long-term risks, and alignment with patients’ preferences. Physicians must now integrate clinical history and physical examination with imaging, laboratory values, and probabilistic models, a cognitive load demanding both critical judgment and scientific literacy.

The pace of change has also accelerated. Whereas new knowledge once took an average of 17 years to reach textbooks and guidelines in the early 2000s, discoveries are now rapidly adopted into practice [9]. This acceleration creates a new challenge: managing the exponential growth of medical knowledge that now unfolds faster than the human capacity for absorption.

## The Anatomy of Medical Thinking: From Gut Feelings to Organic Neural Networks

Unlike a purely logical or algorithmic process, clinical reasoning is dynamic, shaped by perception and inference. It develops in layers, each influenced by internal factors such as knowledge, bias, and emotion and external factors such as context, time, and access to resources. Even before a patient speaks, the clinical gaze captures unstructured information, such as how the patient enters the room or their posture when sitting. This first impression exists at the intersection of intuition and technique, where every detail, no matter how subtle, becomes a potential piece in the diagnostic puzzle.

Then, history taking unfolds as a guided dialogue. Each question refines emerging hypotheses, with the physician adjusting in real time to what is said and what is left unsaid [10,11]. This process goes beyond cataloging symptoms; it involves understanding how the patient perceives their condition within the narrative of their life. Because human behavior is complex and motivations are not binary, accounts may be exaggerated; minimized; or reshaped by fear, shame, or secondary gain. Therefore, the clinician must interpret language, silences, and inconsistencies critically, recognizing that each may alter the trajectory of reasoning.

After anamnesis and physical examination, the delicate process of synthesis begins. Physicians must balance 2 extremes: reasoning too broadly opens up endless possibilities, leading to irrelevant hypotheses, unnecessary tests, and unwarranted interventions, whereas narrowing too quickly risks dismissing critical diagnoses. Recognizing uncertainty is essential as biology is seldom exact. The medical mind operates within layers of probability and judgment, constantly reassessing hypotheses as new information emerges, considering both the plausibility of each possibility and the potential impact of diagnostic errors.

Refining this process requires metacognition: the capacity to monitor one’s own thinking. Although fundamental, it is often neglected in clinical practice [12]. Biases are intrinsic to human reasoning. Anchoring traps thought in the first plausible diagnosis, availability exaggerates the weight of recently observed or common diseases, and confirmation bias narrows attention to evidence that supports an initial hunch [13]. Therefore, structured cognitive models and deliberate reflection are essential safeguards, helping physicians recognize and resist these tendencies.

Clinical decision-making is also shaped by forces beyond the individual. Time constraints, fatigue, and cognitive overload can favor speed over accuracy, impairing analytical depth, whereas clinical settings impose distinct demands: rapid stabilization in the emergency room or slower, narrative-rich deliberation in the clinic. In every case, physicians must balance evidence with the patient’s context, preferences, and values, revealing that clinical reasoning is never a purely technical exercise but a human negotiation [14].

Thus, the greatest challenge to medical reasoning is not AI itself but the stagnation of cognitive training. Physicians are taught protocols and guidelines, which prescribe “what to do,” but receive little formal training in “how to think” in the face of uncertainty [13]. As a result, medicine continues to rely on heuristics and experience even as modern complexity demands structured frameworks for risk interpretation, trade-off management, and adaptive reasoning.

## The Impact of AI on Medical Thinking: Cognitive Expansion or Reduction?

AI has been part of medical practice for decades, from specialized diagnostic systems to ML algorithms that analyze vast clinical, genomic, and epidemiological datasets to uncover hidden patterns [15,16]. In radiology, DL techniques such as convolutional neural networks improve diagnostic accuracy by detecting subtle anomalies that may elude even experienced clinicians [17]. Meanwhile, ML-based models strengthen real-time disease surveillance by processing structured data streams such as case notifications and hospital admissions [18].

However, a turning point emerged with the widespread adoption of LLMs. Unlike earlier predictive algorithms that operated within rigid, preprogrammed parameters and required structural inputs, LLMs interact through natural language, making them accessible for daily clinical tasks such as case analysis and documentation [19]. This ease of use shifts AI from background support to direct influence on decision-making, a change that demands careful scrutiny [20].

Despite their enhanced capacities, LLMs do not “think” in the way humans do. In fact, LLMs recognize patterns, calculate probabilities, and predict the most statistically probable outcome based on previous data. While this can resemble clinical reasoning, it lacks causal inference; they do not ask “why” something happens [21]. Clinical reasoning, in contrast, is adaptive and prospective, integrating mechanisms, trade-offs, and patient narratives to generate and prioritize hypotheses.

This distinction is fundamental in medicine as a model cannot reassess its own assumptions or correct embedded biases. When training datasets contain distortions due to historical disparities, underrepresentation, or flawed methodologies, LLMs amplify these inaccuracies, presenting biased outputs as statistical truth. For instance, a widely used US algorithm underestimated the health needs of Black patients by equating health care cost with health status, thus favoring White patients for additional care [22]. Similarly, generative pretrained transformers have been shown to generate racially and gender-biased recommendations when presented with identical clinical cases, further deepening global health inequities [23].

Clinical reasoning is not just retrospective but adaptive, especially in resource-limited or culturally specific settings. Physicians adjust decisions as evidence and constraints evolve, whereas LLMs remain tied to preexisting data [24]. Consequently, AI trained on generalized datasets may miss local realities such as limited access to medications, diagnostic tools, and specialized care or cultural barriers. For instance, a model might recommend a colonoscopy for iron deficiency anemia in a rural area, overlooking prohibitive costs or reluctance toward invasive procedures. A clinician, aware of these factors, may instead pursue noninvasive tests or negotiate feasible next steps with the patient.

Another limitation is AI’s inability to assimilate unrecorded or deliberately adjusted elements as clinical reasoning relies on subtle cues that often go unregistered in formal records [15]. Compounding this, LLMs often present responses with an unwarranted level of confidence, creating an illusion of precision, a phenomenon called “hallucination” [19]. In contrast, experienced physicians recognize that uncertainty is intrinsic to clinical reasoning, continuously refining hypotheses as new information emerges.

Recognizing these pitfalls, leading regulators have begun to shape “good practices” of AI use in health care. The European Union’s AI Act (2024/1689) designates LLM-based clinical support tools as high-risk devices, mandating data governance, public model documentation, and continuous postmarket surveillance to catch adaptive drift [25]. In parallel, the US Food and Drug Administration’s guidance principles require developers of AI-enabled medical software to specify in advance how and within what limits the model may evolve after approval and demonstrate that such updates will not erode safety or performance [26]. Both frameworks insist on (1) robust quality management to ensure the representativeness and traceability of the training data; (2) meaningful explainability so that clinicians and patients can understand a model’s logic and limitations; and (3) empowering human oversight, signaling a shift toward emerging requirements for the safe and ethical deployment of clinical AI.

## The Cognitive Stethoscope: How to Hear Across AI’s Patterns

The integration of LLMs into medical practice is increasingly likely. However, their impact will depend less on technical capacity than on how physicians choose to engage with them. The danger is not simply that AI can make mistakes but that physicians may stop thinking critically, failing to question, refine, or override their suggestions when necessary. Nonetheless, when properly used, AI can strengthen medical reasoning, serving as a strategic tool to organize thought processes, reduce biases, and validate hypotheses while preserving human judgment (Table 1).

Consider a scenario of a patient with vague abdominal pain. A physician, facing the constraints we have already discussed, may lean toward a common diagnosis such as gastritis and might miss rare but serious conditions. An LLM, drawing from a broader context, could flag mesenteric ischemia as a possibility. While the model cannot determine causality, its input may prompt the physician to revisit the case; order a critical test; and, ultimately, save the patient from a missed diagnosis.

**Table 1.** Pitfalls and successes in physicians’ uses of artificial intelligence (AI) for clinical reasoning.

Aspect	Pitfalls	Mitigation	Successes
Initial assessment	Neglect of clinical cognitive skills such as perception and inference due to premature reliance on AI	Emphasize clinical observation training and delay AI consultation	Enhances data collection by structuring initial patient information efficiently
Hypothesis formation	Overreliance on AI-generated differential diagnoses, potentially overlooking emergent situations or specific sociocultural contexts	Cross-check AI output with situational context and patient narrative	Expands differential diagnosis lists, reducing premature closure and anchoring bias
Decision-making	Passive acceptance of AI recommendations without critical evaluation	Use structured decision frameworks that require justification of AI suggestions	Provides up-to-date evidence and guideline-based recommendations to support decisions
Error handling	Overconfidence in AI outputs due to training data biases or lack of uncertainty calibration	Mandate local calibration studies and introduce uncertainty estimates in outputs	Identifies inconsistencies, flags potential errors, and reduces biases
Patient engagement	Risk of depersonalization by automating interactions with patients or overuse during consultation	Define “AI-free” phases in consultation to focus on human connection	Automates administrative tasks, allowing for more time for meaningful patient interactions
Longitudinal patient management	Fragmented decision-making due to AI operating on isolated encounters	Integrate AI tools with electronic health records to enable continuity-aware insights	Reveals longitudinal patterns that might be missed in episodic human reasoning
Clinical teaching and medical training	Reduced development of diagnostic intuition in trainees due to AI overuse	Balance AI use with mandatory non-AI problem-solving sessions in medical education	Functions as an adaptive learning tool, offering case-based scenarios, interactive feedback, and personalized study recommendations
Ethical decision-making	Inadequate integration of patient values or ethical nuances into AI-driven outputs	Incorporate shared decision-making tools and human review to contextualize ethical trade-offs	Summarizes ethical frameworks, guidelines, and case precedents to aid in ethical deliberation
AI in emergency and high-stakes situations	Delayed or misguided responses in emergencies due to insufficient data for novel cases	Establish protocols where human judgment overrides AI in high-stakes or novel settings	Enhances rapid triage and alerts in time-sensitive, high-risk clinical scenarios

In practice, AI should contribute to clinical reasoning in distinct yet complementary ways. It can rapidly analyze vast datasets to highlight potential diagnoses, suggest additional differential diagnoses, and flag atypical findings that might be overlooked. However, these benefits depend on integration. A recent study showed that an LLM outperformed physicians on a structured diagnostic task when used independently, but embedding it in real-time workflows did not improve diagnostic performance compared to conventional resources, likely due to suboptimal prompting, lack of training, or poor alignment with clinicians’ existing habits [20].

Therefore, physicians remain central. They must deliberately decide which AI-generated insights warrant further pursuit, weighing severity, likelihood and the patient’s context, narrative, and environment. Structured decision-making frameworks can support this process by prompting clinicians to actively justify or question AI suggestions. While algorithms can model probabilities and outcomes, only physicians can interpret them against ethical, contextual, and practical considerations.

While these cautions are essential, they should not obscure the settings in which AI may extend cognitive reach. In low-resource environments, where subspecialty expertise or advanced diagnostics are scarce, AI can broaden differential diagnoses and guide pragmatic next steps. In medical education, it can serve as a scaffold for reasoning, prompting students to articulate hypotheses, test alternatives, and reflect on biases. Beyond these domains, AI can also reinforce safety checks by flagging drug interactions, contraindications, or unintended side effects that warrant monitoring, such as postural hypotension after antihypertensive therapy, prompting proactive follow-up [27].

In ethically sensitive decisions, pairing AI recommendations with shared decision-making tools and explicit human oversight may ensure that care remains aligned with the values and expectations of each patient. Predefined override protocols can be established for situations in which AI may struggle, such as novel presentations or emergencies, reinforcing that human judgment is the final authority in clinical care.

AI can also relieve administrative burdens. AI-powered tools such as ambient documentation systems have shown potential to reduce documentation time for certain subgroups of clinicians, such as those in family medicine [28]. Although these systems have not yielded broad efficiency gains across all users in previous studies, they suggest that AI can meaningfully improve workflow in selected contexts, particularly when adopted with consistent engagement. When linked to longitudinal patient data, AI can further support continuity and coherence, helping decisions extend beyond episodic encounters. This frees time and cognitive bandwidth, allowing physicians to refocus on what technology cannot replace: attentive listening, understanding, and the therapeutic relationship.

Far from replacing the human element, AI can be an ally in restoring the focus on what has always been essential in medicine: the connection between physician and patient. The real opportunity lies in integrating it strategically as a tool for critical reflection. Doubt must remain central to the physician-AI interaction as the physician of the future will not be the one who merely consults AI for answers but the one who knows when to trust it; when to challenge it; and, most importantly, when to think beyond it.

## Conclusions

No previous innovation has had the potential to reshape medical cognition as profoundly as LLMs. To use AI as a cognitive stethoscope is to hear patterns more clearly while never mistaking the instrument for the act of listening itself. The path forward demands deliberate strategies. Clinicians must cultivate metacognitive habits to question and refine AI outputs; educators should embed AI into training as a scaffold for reasoning, prompting learners to articulate hypotheses, test alternatives, and confront biases; and policymakers and developers must ensure transparency, safety, and equity in AI design, with oversight mechanisms to guard against adaptive drift and systemic bias. Future inquiry should examine how AI influences clinical reasoning in real-world practice, evaluate its role in low-resource environments where expertise is scarce, and create regulatory frameworks that adapt alongside evolving models. Only then will it be clear whether AI strengthens cognition or quietly reshapes it in unintended ways.

## Abbreviations

AI: artificial intelligence
DL: deep learning
LLM: large language model
ML: machine learning

## References

[1] Clusmann J, Kolbinger FR, Muti HS (2023). The future landscape of large language models in medicine. Commun Med (Lond).

[2] (2024). FDA digital health and artificial intelligence glossary – educational resource. U.S. Food & Drug Administration.

[3] McDuff D, Schaekermann M, Tu T (2025). Towards accurate differential diagnosis with large language models. Nature.

[4] Hope B (1998). The greatest benefit to mankind: a medical history of humanity from antiquity to the present. BMJ.

[5] Tulchinsky TH (2018). John Snow, cholera, the Broad Street pump; waterborne diseases then and now. Case Stud Public Health.

[6] Sakai T, Morimoto Y (2022). The history of infectious diseases and medicine. Pathogens.

[7] Barberis I, Bragazzi NL, Galluzzo L, Martini M (2017). The history of tuberculosis: from the first historical records to the isolation of Koch’s bacillus. J Prev Med Hyg.

[8] Trickey A, Sabin CA, Burkholder G (2023). Life expectancy after 2015 of adults with HIV on long-term antiretroviral therapy in Europe and North America: a collaborative analysis of cohort studies. Lancet HIV.

[9] Morris ZS, Wooding S, Grant J (2011). The answer is 17 years, what is the question: understanding time lags in translational research. J R Soc Med.

[10] Duffy FD (1998). Dialogue: the core clinical skill. Ann Intern Med.

[11] Helman CG (1981). Disease versus illness in general practice. J R Coll Gen Pract.

[12] Marcum JA (2012). An integrated model of clinical reasoning: dual‐process theory of cognition and metacognition. J Eval Clin Pract.

[13] Corrao S, Argano C (2022). Rethinking clinical decision-making to improve clinical reasoning. Front Med (Lausanne).

[14] Stewart M, Brown JB, Weston W, McWhinney IR, McWilliam CL, Freeman T (2013). Patient-Centered Medicine: Transforming the Clinical Method.

[15] Giordano C, Brennan M, Mohamed B, Rashidi P, Modave F, Tighe P (2021). Accessing artificial intelligence for clinical decision-making. Front Digit Health.

[16] Eloranta S, Boman M (2022). Predictive models for clinical decision making: deep dives in practical machine learning. J Intern Med.

[17] Yamashita R, Nishio M, Do RK, Togashi K (2018). Convolutional neural networks: an overview and application in radiology. Insights Imaging.

[18] Wiens J, Shenoy ES (2018). Machine learning for healthcare: on the verge of a major shift in healthcare epidemiology. Clin Infect Dis.

[19] Thirunavukarasu AJ, Ting DS, Elangovan K, Gutierrez L, Tan TF, Ting DS (2023). Large language models in medicine. Nat Med.

[20] Goh E, Gallo R, Hom J (2024). Large language model influence on diagnostic reasoning: a randomized clinical trial. JAMA Netw Open.

[21] Stead WW (2018). Clinical implications and challenges of artificial intelligence and deep learning. JAMA.

[22] Obermeyer Z, Powers B, Vogeli C, Mullainathan S (2019). Dissecting racial bias in an algorithm used to manage the health of populations. Science.

[23] Zack T, Lehman E, Suzgun M (2024). Assessing the potential of GPT-4 to perpetuate racial and gender biases in health care: a model evaluation study. Lancet Digit Health.

[24] Gao Y, Myers S, Chen S (2025). Uncertainty estimation in diagnosis generation from large language models: next-word probability is not pre-test probability. JAMIA Open.

[25] (2024). Regulation (EU) 2024/1689 of the European Parliament and of the Council of 13 June 2024 laying down harmonised rules on artificial intelligence and amending Regulations (EC) no 300/2008, (EU) no 167/2013, (EU) no 168/2013, (EU) 2018/858, (EU) 2018/1139 and (EU) 2019/2144 and Directives 2014/90/EU, (EU) 2016/797 and (EU) 2020/1828 (Artificial Intelligence Act) (text with EEA relevance). European Union.

[26] (2018). Software as a Medical Device (SaMD). U.S. Food & Drug Administration.

[27] Choudhury A, Asan O (2020). Role of artificial intelligence in patient safety outcomes: systematic literature review. JMIR Med Inform.

[28] Liu TL, Hetherington TC, Dharod A (2024). Does AI-powered clinical documentation enhance clinician efficiency? A longitudinal study. NEJM AI.

